# Clinical impact of a cytological screening system using cyclin D1 immunostaining and genomic analysis for the diagnosis of thyroid nodules

**DOI:** 10.1186/s12885-019-5452-4

**Published:** 2019-03-18

**Authors:** Masanori Teshima, Kazuya Tokita, Eijitsu Ryo, Fumihiko Matsumoto, Madoka Kondo, Yota Ikegami, Hirotaka Shinomiya, Naoki Otsuki, Nobuyoshi Hiraoka, Ken-ichi Nibu, Seiichi Yoshimoto, Taisuke Mori

**Affiliations:** 1 Department of Pathology, National Cancer Center Hospital, Tokyo, Japan; 2 Division of Molecular Pathology, National Cancer Center Research Institute, Tokyo, Japan; 3 Department of Head and Neck Surgery, National Cancer Center Hospital, Tokyo, Japan; 4 Department of Otolaryngology – Head and Neck Surgery, Kobe University, School of Medicine, Kobe, Japan

**Keywords:** Thyroid nodule, Diagnosis, Cytology, Cyclin D1, NGS

## Abstract

**Background:**

Fine-needle aspiration (FNA) is the most reliable method for diagnosing thyroid nodules; however, some features such as atypia of undetermined significance or follicular lesion of undetermined significance can confound efforts to identify malignancies. Similar to BRAF, cyclin D1 may be a strong marker of cell proliferation.

**Methods:**

One hundred two patients with thyroidal nodule were enrolled in this prospective study.

Expression of cyclin D1 in thyroid nodules was determined by immunohistochemistry using both surgical specimens and their cytological specimens. The identification of the optimal cut off points for the diagnosis of malignancy were evaluated using the receiver operating characteristic (ROC) curves and the assessment of the area under the ROC curve (AUC). The specificity, sensitivity, positive predictive value (PPV) of markers were evaluated from crosstabs based on cut off points and significance were calculated. We also analyzed genetic variants by target NGS for thyroid nodule samples.

**Results:**

The positive predictive value (PPV) and median stain ratio (MSR) of cyclin D1 nuclear staining was determined in papillary thyroid carcinoma (PPV = 91.5%, MSR = 48.5%), follicular adenoma (PPV = 66.7%, MSR = 13.1%), and adenomatous goiter and inflammation controls (MSR = 3.4%). In FNA samples, a threshold of 46% of immunolabelled cells allows to discriminate malignant lesions from benign ones (*P* < 0.0001), with 81% sensitivity and 100% specificity. A 46% cutoff value for positive cyclin D1 immunostaining in thyroid cells demonstrated 81% sensitivity and 100% specificity. In surgical specimens, ROC curve analysis showed a 5.8% cyclin D1 immunostaining score predicted thyroid neoplasms at 94.4% sensitivity and 92.3% specificity (*P* = 0.003), while a 15.7% score predicted malignancy at 86.4% sensitivity and 80.5% specificity (*P* < 0.0001). Finally, three tested clinico-pathological variables (extra thyroidal extension, intraglandular metastasis, and lymph node metastasis) were significant predictors of cyclin D1 immunostaining (*P* < 0.001).

**Conclusion:**

Our cytological cyclin D1 screening system provides a simple, accurate, and convenient diagnostic method in precision medicine enabling ready determination of personalized treatment strategies for patients by next generation sequencing using cytological sample.

**Electronic supplementary material:**

The online version of this article (10.1186/s12885-019-5452-4) contains supplementary material, which is available to authorized users.

## Background

Thyroid nodules are defined as nodules originally detected in a patient with no thyroid-related clinical symptoms, examination findings, or suspected thyroid disease. These nodules are most commonly detected from medical imaging procedures, such as computed tomography, magnetic resonance imaging, and positron emission tomography, of the neck [1, 2]. Ultrasound is the most utilized medical imaging procedure for thyroid nodules. Currently, fine-needle aspiration (FNA) is the most reliable method to detect thyroid nodules; however, ultrasound detects solid hypoechoic features that are more likely to be malignant, with a sensitivity and specificity of 80 and 70%, respectively [3]. Malignancy rates in nodules in the cytology category of atypia of undetermined significance/follicular lesion of undetermined significance (AUS/FLUS) are higher than previously estimated, with 26.6–37.8% of AUS/FLUS nodules harboring cancer [4]. In particular, there are no established criteria for the presurgical diagnosis of noninvasive follicular thyroid neoplasms such as a well-differentiated thyroid tumor of uncertain malignant potential, and its distinction from a follicular adenoma and/or classic papillary thyroid carcinoma [5, 6].

Cyclin D1 (also known as CCND1) is a regulator of the G1/S-phase of cell cycle. In early G1 phase, cell synthesis after mitogenic stimulation is crucial in rescuing the cell from the resting G0 state. Cyclin D1, and to a lesser extent the other D-type cyclins, is deregulated in cancer and is a biomarker of cancer phenotype and disease progression [7, 8]. Consistent with its positive effect on cell cycle progression, cyclin D1 is a proto-oncogene frequently overexpressed in many cancers [9, 10]. In addition, one study reported that cyclin D1 contributes to BRAF-inhibitor resistance in cancers such as malignant melanoma [11], whereas other studies found that cyclin D1 is overexpressed in papillary thyroid carcinoma (PTC) [12, 13].

Because of the demonstrated role of cyclin D1 in PTC, we considered the potential clinical use of cyclin D1 immunostaining of preliminary samples of thyroid neoplasm. Thus, the purpose of this study was to evaluate the cyclin D1 immunostaining screening system for thyroid neoplasms as a biomarker for tumor aggressiveness. More specifically, our aim was to examine cyclin D1 immunostaining and analyze the expression profile of genes from FNA samples. In addition, we investigated the prospect of a new preoperative cytological strategy for the detection of thyroid nodules using a combination of cyclin D1 immunostaining and genetic sequence analysis.

## Methods

### Clinical data

This analysis utilized 102 resected thyroid specimens between 2008 and 2017 at the National Cancer Center Hospital, Tokyo, Japan. We obtained tissue samples from thyroid surgery or head and neck surgery for malignant tumor. We prospectively collected 31 thyroid aspirates from the aforementioned 102 patients in liquid-based cytology (LBC; CytoRich Blue Preservative™from BD) from 2015 until 2017 which were formalin-fixed. Therefore, cases of cytology and cases of histology are the same. The 102 thyroidal tumor surgical specimens included 59 papillary carcinomas (PTC), 9 follicular carcinomas (FC), 7 poorly differentiated thyroid carcinomas (PDC), 1 medullary thyroid carcinoma (MTC), 5 anaplastic thyroid carcinomas (ATC), 2 well-differentiated tumors with uncertain malignant potential (WP), 7 follicular adenomas (FA), and Twelve individuals were background (BG) cases: 1 with adenomatous goiter (AG) and 11 with no neoplastic thyroid. BG consisted of normal thyroid tissue such as resections with surgery for hypopharyngeal carcinoma or laryngeal carcinoma.

### Immunohistochemistry for FFPE

Formalin-fixed paraffin-embedded specimens (FFPE) were cut into 10-μm-thick sections. Deparaffinized sections were subjected to hematoxylin–eosin staining, cyclin D1 staining, and Ki67 staining. Immunohistochemical staining was performed with the primary antibodies Cyclin D1 (Ready to use; SP4, Nichirei) and Ki67 (Ready to use; MiB-1, Dako). Each section was exposed to 0.3% hydrogen peroxide for 15 min to block endogenous peroxidase activity. For staining, we used an automated stainer (Dako) according to the manufacturer’s protocol.

### Immunohistochemistry for cytological specimens

Thyroid samples were fixed in CytoRich Blue Preservative™ (BD). Unstained slides were then processed on the PrepStain™ (BD) instrument according to the manufacturer’s protocol. Immunocytochemistory (ICC) was performed on an automated stainer (DAKO) as described in the FFPE materials protocol.

### Tissue microarray

We selected FFPE tissue blocks containing the main tumor area and the normal thyroid area previously used for histological examination. Two 2.0 mm diameter core specimens for each case were taken from these blocks and transferred to recipient blocks using a TMA Master tissue microarray (3DHistech, Budapest, Hungary) [14]. The TMA include 33 thyroid tumors (21 PTC, 5 FC, 3 FA, and 4 ATC).

### Evaluation of immunostaining

Scanned slides were digitized at 20_X_ magnification using the Hamamatsu NDP slide scanner (Hamamatsu Nanozoomer 2.0 HT) and analyzed using its viewing platform (NDP View). The pathologists chose three fields of vision per slide and analysed the areas using the Gunma-LI, Image J software. We utilized automated quantitative analysis for detection of cyclin D1 sensitivity and Ki67 index using Gunma-LI, Image J. Cyclin D1 positive ratio cutoffs from the 50th to the 95th percentile along with their corresponding sensitivity and specificity for diagnosis of malignant lesion vs background and neoplasm vs background were calculated.

### Evaluation of cytology

The specimen was observed at low magnification (× 100) and 6 independent cell clumps with the strongest cyclin D1 expression were selected. Next, the presence of 10 or more follicular epithelium composed of cell clumps was confirmed. At high magnification (× 400), the number of constituent cells of the clump and positive cells were counted using the cell counter. The cyclin D1 positive rate of the cell clump was calculated. Three cytotechnologists independently counted the cells. The 31 thyroid FNA specimens included 12 PTC, 2 PDC, 2WP, 1 MTC, 1 ATC, 3 FA, and 10 individuals were background (BG) cases with no neoplastic thyroid. BG consisted of normal thyroid tissue such as resections with surgery for hypopharyngeal carcinoma or laryngeal carcinoma. After the resection of hypopharynx or larynx, cytotechnologists performed FNA with normal thyroid and preserved LBC immediately.

### DNA extraction from FFPE samples

Deparaffinized 10-μm-thick sections from each paraffin block were microdissected using sterilized toothpicks under a microscope to enrich tumor content. The microdissected samples were subjected to DNA extraction using the GeneRead DNA FFPE Kit (Qiagen, Hilden, Germany). Fixed cells were collected and DNA was extracted using the QIAamp DNA Mini kit (Qiagen, Hilden, Germany).

### DNA Extraction from LBC samples

The LBC fixed cell residue was collected and washed with PBS. DNA was extracted using the QIAamp DNA Micro kit (Qiagen, Hilden, Germany).

### Next-generation sequencing

Next-generation sequencing (NGS) libraries were prepared by two-step tailed PCR. The first PCR round was performed using two pools of primers consisting of a gene-specific sequence and a consensus primer binding sequence. Each pool contained 36 pairs of primers targeting frequently mutated regions of *p53, BRAF, KRAS, HRAS, CTNNB1, AKT1,* and *PI3KCA*. Each PCR reaction was performed using 10 ng genomic DNA with Kapa HiFi Hotstart Ready Mix (Kapa Biosystems, Boston, MA, USA). Amplification conditions were: initial denaturation for 3 min at 95 °C, 23 cycles of 20 s at 98 °C, 20 s at 64 °C, and 20 s at 72 °C, and final extension of 3 min at 72 °C. The PCR products were purified using the Agencourt AMPure XP kit (Beckman Coulter, Brea, CA, USA) and eluted in 30 μl low TE buffer. The second round PCR was performed using 5 μl of purified first round PCR products to incorporate sample-specific indexes and P5/P7 flow-cell binding sequences (Additional file 1: Table S1). Amplification conditions of the second round PCR were: initial denaturation for 3 min at 95 °C, 10 cycles of 20 s at 98 °C, 20 s at 65 °C, and 20 s at 72 °C, and final extension of 3 min at 72 °C. The products were purified and eluted in 30 μl low TE buffer as above. The library concentration was determined by quantitative PCR using KOD SYBR qPCR Mix (Toyobo, Tokyo, Japan) and DNA standards (KK4903; Kapa biosystems) and adjusted to 4 nM. Single-read sequencing was performed using MiSeq (Illumina, San Diego, CA, USA) and MiSeq Reagent Micro Kit v2 (300 cycles) (Illumina) according to the manufacturer’s protocol. The resulting sequences were mapped onto the human reference genome hg19 following removal of primer sequences on each end of amplicons using the TruSeq Amplicon application (Illumina). Sequence variations with variant allele frequencies of more than 5% were identified as candidate mutations. Synonymous mutations and common single nucleotide polymorphisms, based on the Single Nucleotide Polymorphism Database build 137, were excluded.

### Fluorescent in situ hybridization (FISH)

Cyclin D1 FISH analysis was performed within 4-μm-thick FFPE tumor sections. Visis CCND1/CEP 11 (Abott SA, Belgium) was used to test for cyclin D1 amplification. The FISH images were captured using the Metafer Slide Scanning Platform (MetaSystem, Altlussheim, Germany), and 50 non-overlapping tumor cells were examined.

### Statistical analysis

Correlations between the results of immunostaining for cyclin D1 and tumor size, extra thyroidal extension, intraglandular metastasis, lymph node metastasis, and distant metastasis were analyzed statistically using the chi-square test (cutoff level for significance was *p* < 0.05). The diagnostic performances of immunolabeling and the identification of the optimal cutoff points for the diagnosis of malignancy were evaluated using receiver operating characteristic (ROC) curves and the assessment of the area under the ROC curve (AUC). The specificity, sensitivity, and positive predictive value (PPV) of markers were evaluated from crosstabs based on cutoff points and significance was calculated. Statistical analysis were performed with JMP® 13 (SAS institute Inc., Cary, NC, USA).

## Results

### Patients and characteristics

Clinico-pathological characteristics of our recruited cohort (*n* = 102), including age, gender, clinical data, and histopathologic features are shown in Table 1. We found that 19 patients (18.6%) had tumors with a diameter ≥ 4 cm, 57 patients (55.8%) had tumors with an extra thyroidal extension, 26 patients (25.5%) had intraglandular metastasis, 50 patients (49%) had neck lymph node metastasis in the central and lateral compartments, and distant metastasis was noted in 28 patients (27.5%). Follow up periods after treatment ranged from 1 to 168 months (median, 9 months).

**Table 1:** Patient and Tumor Characteristics

	No	%
Age, y, mean	64 (11–85)	
Gender		
Male	39	(37.1)
Female	63	(62.9)
Histological type		
Papillary thyroid carcinoma	59	(57.8)
Medullary thyroid carcinoma	1	(0.9)
Follicular carcinoma	9	(8.8)
Poorly differentiated thyroid carcimoma	7	(6.9)
Well differentiated tumor - uncertain malignant potential	2	(2.0)
Follicular adenoma	7	(6.9)
Anaplastic thyroid carcinoma	5	(4.9)
Background (inflammation or goiter)	12	(11.8)
Tumor Size (mm)		
<40	83	(81.4)
≧40	19	(18.6)
Extrathyroidal extension		
Absent	44	(43.1)
Present	58	(56.9)
Intraglandular metastasis		
Absent	76	(74.5)
Present	26	(25.5)
Lymph node metastasis		
Negative	52	(51)
Positive	50	(49)
Distant metastasis		
Absent	74	(72.5)
Present	28	(27.5)

### Immunohistochemical results

**Fig. 1:**
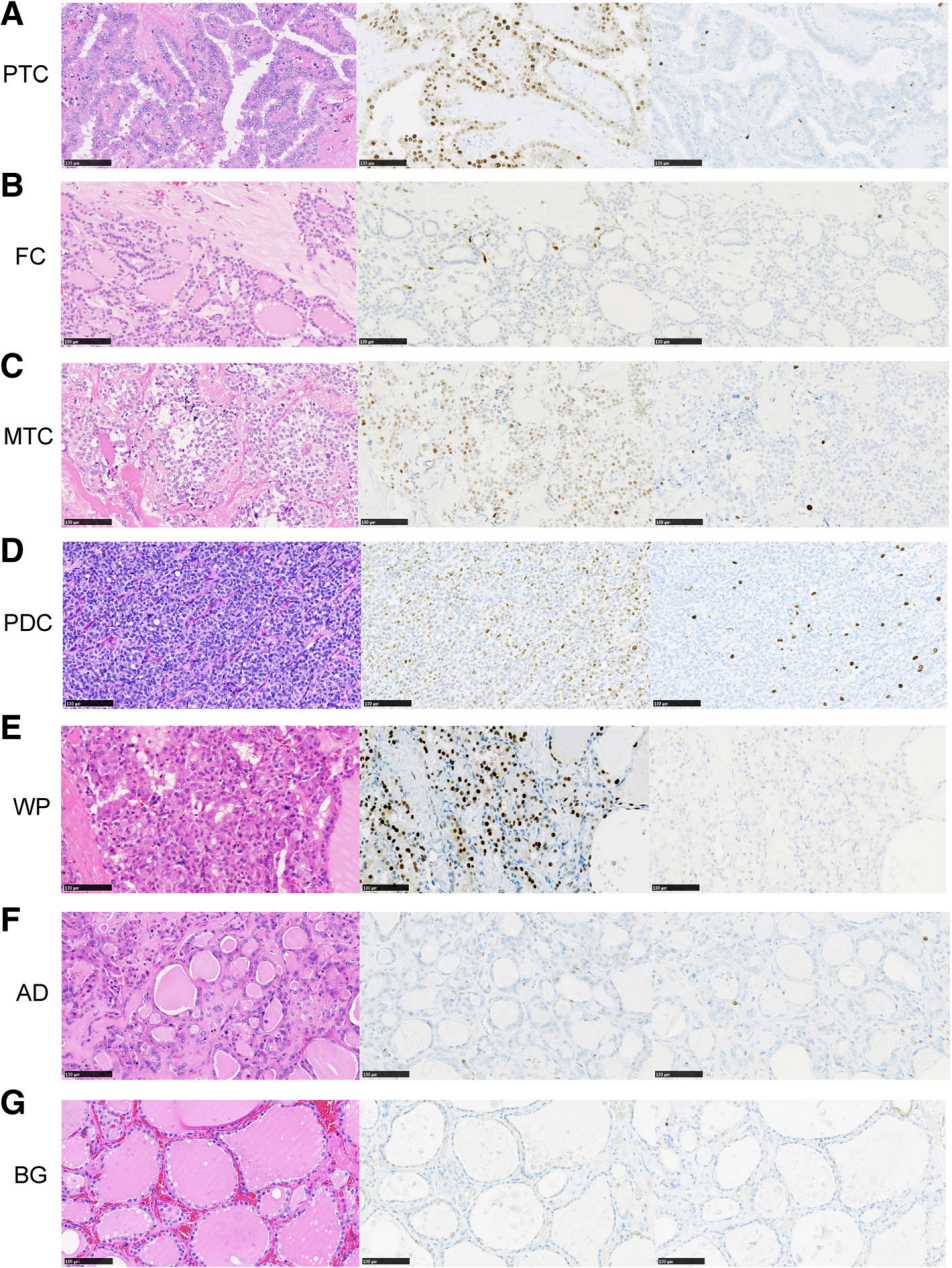
Histological features of thyroid tumors with cyclin D1 and Ki-67 immunostaining. Papillary thyroid carcinoma (**a**: PTC), Follicular carcinoma (**b**: FC), Medullary thyroid carcinoma (**c**: MTC), Poorly differentiated carcinoma (**d**: PDC), Well-differentiated tumor with uncertain malignant potential (E: WP), Adenoma (**f**: AD), and Background (**g**: BG). From the left column, HE, Cyclin D1, ki67 respectively. Scale bar: 100 μm

We evaluated the positive rate of cyclin D1 and Ki-67 in all cases. The subjects were diagnosed with PTC, MTC, FC, PDC, WP, FA, or ATC, and the control was diagnosed with AG or had a non-tumorous thyroid gland. We determined the positive predictive value (PPV) and median stain ratio (MSR) of nuclear cyclin D1 staining in samples from patients with PTC (PPV = 91.5%, MSR = 48.5%), MTC (PPV = 100%, MSR = 66.4%), FC (PPV = 77.8%, MSR = 34.2%), PDC (PPV = 85.7%, MSR = 45.9%), ATC (PPV = 33.3%, MSR = 15%), WP (PPV = 100%, MSR = 41.5%), and FA (PPV = 66.7%, MSR = 13.1%). Samples of controls with BG exhibited a cyclin D1 MSR of 3.4%. In addition, we evaluated the nucleus staining ratio of Ki67 in samples of patients with PTC (PPV = 76.3%, MSR = 3.5%), MTC (PPV = 0%, MSR = 4%), FC (PPV = 11.1%, MSR = 2.4%), PDC (PPV = 28.6%, MSR = 4.1%), ATC (PPV = 66.7%, MSR = 10.4%), WP (PPV = 50%, MSR = 3.2%), FA (PPV = 20%, MSR = 2.3%), and controls with BG (MSR = 3.1%) (Table 2). No immunostaining was found in adjacent normal thyroid and AG tissues (Fig. 1).

**Table 2:** Cyclin D1 and Ki67 immunolabelling in the histological variants of Thyroid neoplasm

	Percentage of Cyclin D1			Percentage of Ki67		
Variants of Thyroid neoplasm	Immunolabelled tumor cells (%)			Immunolabelled tumor cells (%)		
	MSR	Mean ± SD	PPV	MSR	Mean ± SD	PPV
	(Range)			(Range)		
Papillary thyroid carcinoma (*n* = 59)	48.5	42.9 ± 19.1	91.5	3.5	5.2 ± 5.7	76.3
	(6.1–76.7)			(0.2–37.4)		
Medullary thyroid carcinoma (n = 1)	66.4	66.4	100	4	4	11.1
Follicular carcinoma (*n* = 9)	34.2	36.0 ± 26.6	77.8	2.4	4.5 ± 6.1	28.6
	(5.8–76)			(0.9–20.3)		
Poorly differentiated thyroid carcinoma (*n* = 7)	45.9	42.3 ± 21	85.7	4.1	5.4 ± 4.2	60
	(2.8–68.1)			(0.3–12.2)		
Well differentiated tumor-uncertain malignant potential (*n* = 2)	41.5	41.5 ± 3.2	100	3.15	3.2 ± 2.9	50
	(39.2–43.7)			(1.1–5.2)		
Follicular adenoma (n = 7)	13.1	17.5 ± 15.3	66.7	2.3	2.9 ± 1.9	20
	(2.5–43.2)			(1–6.8)		
Anaplastic thyroid carcinoma (n = 5)	9.9	17.2 ± 19.5	33.3	10.4	8.3 ± 3.5	66.7
	(1.9–49.7)			(4.4–11.7)		
Background (inflammation or adenomatous goiter, *n* = 12)	3.4	3.4 ± 0.9		3.05	3.7 ± 1.4	
	(2.1–5.2)			(2.1–7.2)		

*MSR Medisan stain ratio, *PPV* Positive predictive value

**Fig. 2:**
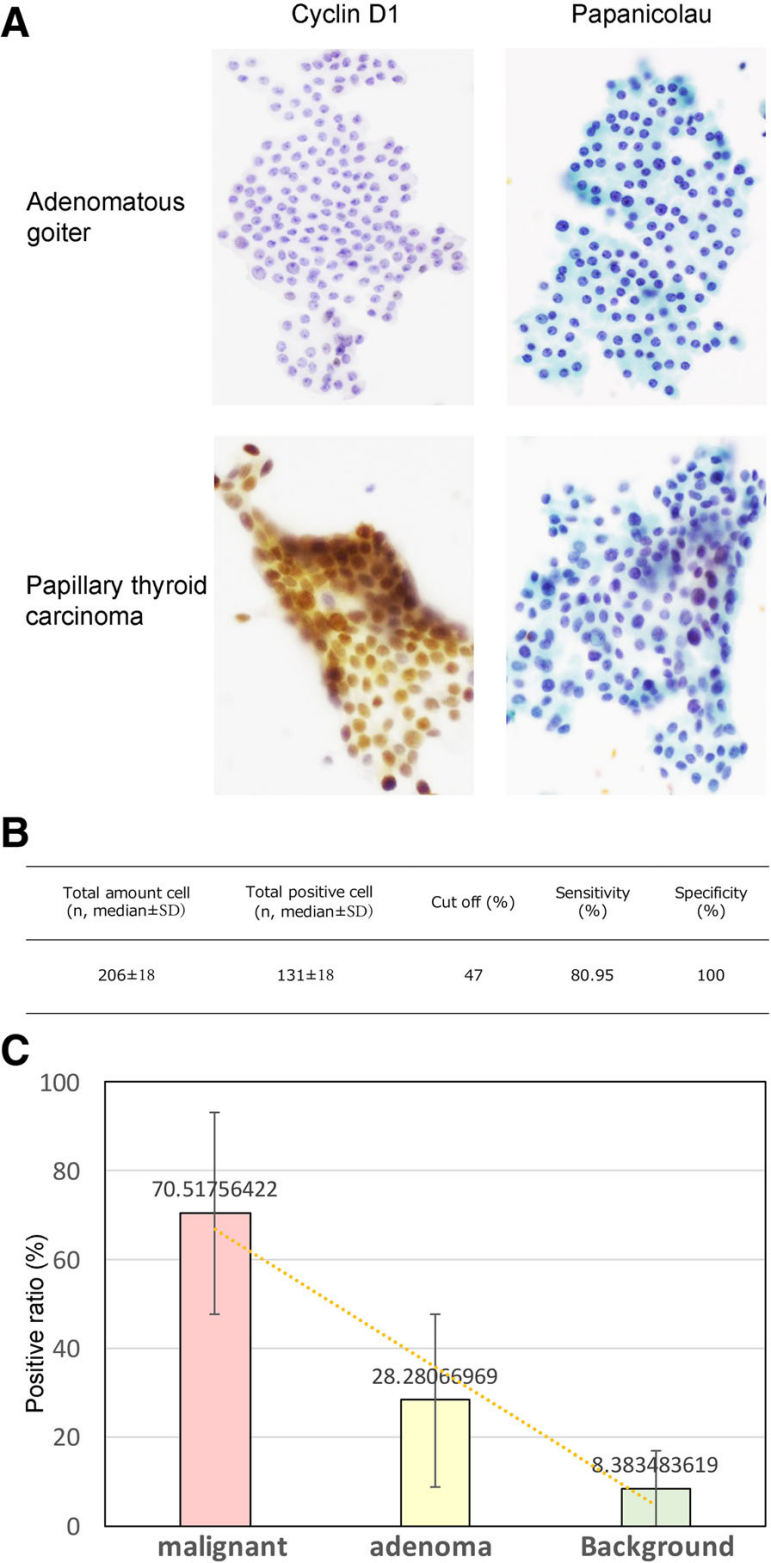
Cytological cyclin D1 immunostaining of thyroid neoplasms. **a** Cytological features of adenomatous goiter and papillary thyroid carcinoma using cyclin D1 immunostaining and Papanicolaou stain. Note absence of cyclin D1 immunostaining in adenomatous goiter and positive nuclear staining for cyclin D1 in papillary thyroid carcinoma. **b** Cytology of cyclin D1 immunolabeling with different types of thyroid neoplasms (*n* = 31). **c** There is correlation between cyclin D1- nuclear expression and tumor malignancy in thyroid neoplasm cells

For our cytology analysis, we determined adequate samples as follows: at least six groups of well-preserved follicular cells (10 or more cells per group), six groups of follicular cells on at least two slides from separate passes, and a minimum of 10 clusters of follicular cells. We also used LBC to investigate cyclin D1 immunolabeling in our cohort with thyroid neoplasms. Using this approach, we found that each sample contained a median value of 206 cells in total, of which a median of 131 cells were positive for cyclin D1 immunostaining with a mean positive ratio of 61% per sample. Using a nuclear cyclin D1 immunostaining proportion of the cut-off threshold for malignant thyroid neoplasm positivity from thyroid neoplasm was set at 46%, we found that cyclin D1 positivity in FNA samples was significantly associated with histologic type (*P* < 0.0001). Using a threshold of 46% of immunolabelled cells allowed to significantly discriminate malignant thyroid neoplasms from benign lesions in FNA samples. This threshold showed an 85% sensitivity and 100% specificity (Fig. 2 and Table 3).

**Table 3:** ROC Curve to Confirm Cyclin D1 Immunostaining Cutoff Threshold

Histology					
Immunolabelling cells	Percentage of Cyclin D1 immunolabelled tumor cells				
	Cut off (%)	AUC	p	Sensitivity (%)	Specificity (%)
Carcinoma VS Adenoma/Background	15.7	0.89	<.0001*	86.4	80.5
Carcinoma/Adenoma VS Background	5.8	0.93	0.003*	94.4	92.3
Immunolabelling cells	Percentage of Ki67 immunolabelled tumor cells				
	Cut off (%)	AUC	p	Sensitivity (%)	Specificity (%)
Carcinoma VS Adenoma/Background	3.5	0.58	NS	53.1	71.4
Carcinoma/Adenoma VS Background	5	0.54	NS	33.7	92.3
Cytology					
Immunolabelling cells	Percentage of Cyclin D1 immunolabelled tumor cells				
	Cut off (%)	AUC	p	Sensitivity (%)	Specificity (%)
Malignant VS Adenoma/Background	46	0.96	<.0001*	85	100

### ROC curve to confirm cyclin D1 immunostaining cutoff threshold

Through ROC curve analysis, we found that the cyclin D1 immunostaining score at 5.8% identified thyroid neoplasms with a sensitivity of 94.4% and specificity of 92.3% in histological analysis (*P* = 0.003). The area under the curve was 93.1%. We also found that the immunostaining score of cyclin D1 at 15.7% predicted a thyroid malignancy with a sensitivity of 86.4% and specificity of 80.5% in histological analysis (*P* < 0.0001) (Table 3).

### Relationship between cyclin D1 expression and Clinico-pathological variables

Next, we evaluated the relationship between cyclin D1 expression and different clinico-pathological characteristics based on the determined cutoff point. We divided our patient cohort into two groups based on maximum tumor diameter [15]. Eighty-three patients (81.4%) had tumors < 4 cm maximum diameter, while 19 patients (18.6%) had tumors ≥4 cm. Twenty-eight patients with thyroid cancer had distant metastases. After thyroidectomy, histopathological analysis of the thyroid showed an extra thyroidal extension of the tumor in 57 patients (55.9%). Intraglandular metastasis occurred in 26 patients (25.5%), in which both lobes of the gland were affected, and capsular infiltration was observed in 29 cases (58%). Of these five evaluated parameters, we found that extra thyroidal extension, intraglandular metastasis, and lymph node metastasis were significant predictors of cyclin D1 immunostaining (all *P* < 0.0001). Other parameters were not significantly correlated (Table 4).

**Table 4:** Correlation between Cyclin D1 immunoreactivity and clinico-pathological data

Paramater	Cyclin D1 (Immunolabeling ratio≧ 15.7%)				*p*
	Immunolabeling below threshold		Immunolabeling above threshold		
Tumor size (mm)					
<40	15	14.70%	68	66.70%	0.59
≧40	3	2.90%	16	15.70%	
Extra-thyroidal extension					
Present	3	2.90%	54	52.90%	0.0002^*^
Absent	15	14.70%	30	29.40%	
Intraglandular metastasis					
Present	0	0.00%	26	25.50%	0.0006^*^
Absent	18	17.70%	58	56.90%	
Lymph node metastasis					
Positive	1	1.00%	49	48.00%	<.0001^*^
Negative	17	16.70%	35	34.30%	
Distant metastasis					
Present	4	3.90%	24	23.50%	0.6
Absent	14	13.70%	60	58.80%	

*: significant

### FISH results

We found no evidence of *CCND1* copy number change in any cases, which included 35 thyroidal tumors (PTC:21, FC: 5, FA: 3, ATC: 4, and WP: 2) (data not shown).

### NGS findings

Table 5 summarizes our comparison of genetic variants identified by targeted NGS in LBC and FFPE samples of thyroid tumors. We performed genetic analysis of three cases using available sequence data from LBC and FFPE samples and found that the *BRAF* V600E mutation was detected in two cases and mutations in *HRAS* and *TP53* were detected in one case. There was no difference in detecting genetic changes in sequencing data obtained from LBC and FFPE samples.

**Table 5:** Comparison of genetic variants identified by target NGS in LBC and FFPE samples of thyroid tumors

Patient no	Gene	cDNA change	Amino acid change	Variant frequency (%)	Read depth	Functional consequence
Case 29 LBC	HRAS	c.81A > G		50	104	synonymous variant
	TP53	c.215G > C	p.72P/R	99.66	1468	missense variant
Case 29 FFPE	HRAS	c.81A > G		48.75	80	synonymous variant
	TP53	c.215G > C	p.72P/R	98.86	612	missense variant
Case 30 LBC	BRAF	c.1799A > T	p.600 V/E	31.98	172	missense variant
Case 30 FFPE	BRAF	c.1799A > T	p.600 V/E	2.87	348	missense variant
Case 31 LBC	BRAF	c.1799A > T	p.600 V/E	40.46	351	missense variant
Case 31 FFPE	BRAF	c.1799A > T	p.600 V/E	25.68	183	missense variant

*LBC* liquid based cytology sample, *FFPE* formalin fixed paraffin embedded specimen

## Discussion

Although ultrasound-guided FNA biopsy is widely used to investigate nonpalpable thyroid nodules, the goal of diagnosis is to precisely define whether there is a need to resect or for active surveillance [3]. Implementation of the Bethesda System for Reporting Thyroid Cytopathology has improved the quality of FNA reporting, promoting greater transparency and fewer unwarranted thyroidectomies [16]. The AUS/FLUS category, known as Bethesda Category III, has been ascribed a malignancy risk of 5–15%, but the probability of malignancy in AUS/FLUS specimens remains unclear [4]. An atypical cell of undetermined diagnosis (ACUS) would be used in situations such as a sparsely cellular aspirate with a predominance of microfollicles, cytologic atypia in the setting of preparation artifact, a mixed cytoarchitectural pattern that includes nearly equal proportions of macrofollicles and microfollicles, and focal atypia suggestive of papillary carcinoma in an otherwise predominantly benign-appearing sample [17].

Diagnostically, most thyroid aspirations represent benign colloid nodules. The quantity of colloid versus the number of cells is often the most important diagnostic finding [18]. FNA is frequently complicated by aspiration of blood, particularly in vascular organs like the thyroid, which compromises cellular preservation and interpretation. Furthermore, many diagnostic pitfalls exist in the interpretation of thyroid specimens making excellence of cellular material a prerequisite for reliable diagnosis. Based on our findings in the current study, we found that cyclin D1 immunostaining provided a powerful, robust cytology-based thyroid diagnosis.

The cyclin D1 oncogene encodes the regulatory subunit of a holoenzyme that phosphorylates and inactivates Rb protein and promotes progression through the G1 to S phase of the cell cycle [19]. Cyclin D1 is also frequently deregulated in cancer and is a biomarker of cancer phenotype and disease progression. The ability of cyclins to activate the cyclin-dependent kinases CDK4 and CDK6 is the most extensively documented mechanism for their oncogenic action and provides an attractive therapeutic target [7]. Previously, it was reported that tumor mechanisms of primary resistance include mutations in *RAC1*, loss of *PTEN*, and copy number increase of *CCND1*, while secondary resistance mechanisms include alternative expression of *BRAF* and *PI3K* [20]. Therefore, we focused on evaluating cyclin D1 immunostaining in thyroid neoplasms. Cyclin D1 is correlated with various tumor features, and the accumulation of cyclin D1 protein in the nucleus, detected by immunohistochemistry, is suggested as an adverse risk factor in various tumors, including prostate cancer [9] and lung cancer [21]. Some studies reported that cyclin D1 overexpression significantly correlates with thyroid tumors [13, 22]. Similarly, Ki67 is used as a measure of tumor proliferation, particularly in breast cancer [23]. Although copy number changes in *CCND1* were not detected by FISH in samples of malignant thyroid tumors in our study, we identified cyclin D1 immunostaining for diagnosis of thyroid neoplasm because of its high sensitivity and specificity compared with Ki67 immunostaining. Furthermore, we determined a cutoff limit (diagnostic threshold) based on our ROC curve analysis. We found cyclin D1 immunostaning with adenoma and malignant subtypes and absence of immunostaining with normal thyroid and AG. We also found that cyclin D1 had greater sensitivity and specificity than Ki67. Based on these findings, we posit that differentiated thyroid carcinoma has slow cell proliferation; however, several ATC showed random staining patterns and low levels of cyclin D1. ATC is characterized by a rapid rate of cellular proliferation and de-differentiation [23, 24]. We speculate that there are differences in cell chromosomal instability between ATC and other thyroid carcinomas types. We defined the diagnosis of poorly differentiated carcinoma based on criteria from a previous study [25]. Similarly, a few cases of PDC have varied cyclin D1 immunostaining intensities.

On the other hand, AG and normal thyroid tissue were clearly separated by cyclin D1 in our cytological analysis system. As a further advantage, Ki67 positively stained lymphocytes but cyclin D1 did not [8, 26]. Moreover, in our clinico-pathological analysis, we found the rate of regional lymph node metastasis, extrathyroidal extension, and intraglandular metastasis significantly correlated with increased cyclin D1-immunostaining. This association between cyclin D1 overexpression and LN metastases in thyroid neoplasm is concordant with a previous report [22]. Thus, the level of cyclin D1 overexpression may be important parameters in estimating tumor growth and metastatic potential of thyroid malignant tumors towards clinical features.

In this study, we determined that thyroid neoplasms showed cytological immunostaining of cyclin D1 with six independent cell clumps based on the Bethesda System. By integrating histological observations with mutational analysis of samples used for cytological diagnosis, we may be able to not only identify neoplastic lesions, but also discriminate between benign or malignant tumors and determine the sub-type. Mutations in *BRAF* and *RAS* are well characterized in thyroid cancer [27–29].

**Fig. 3:**
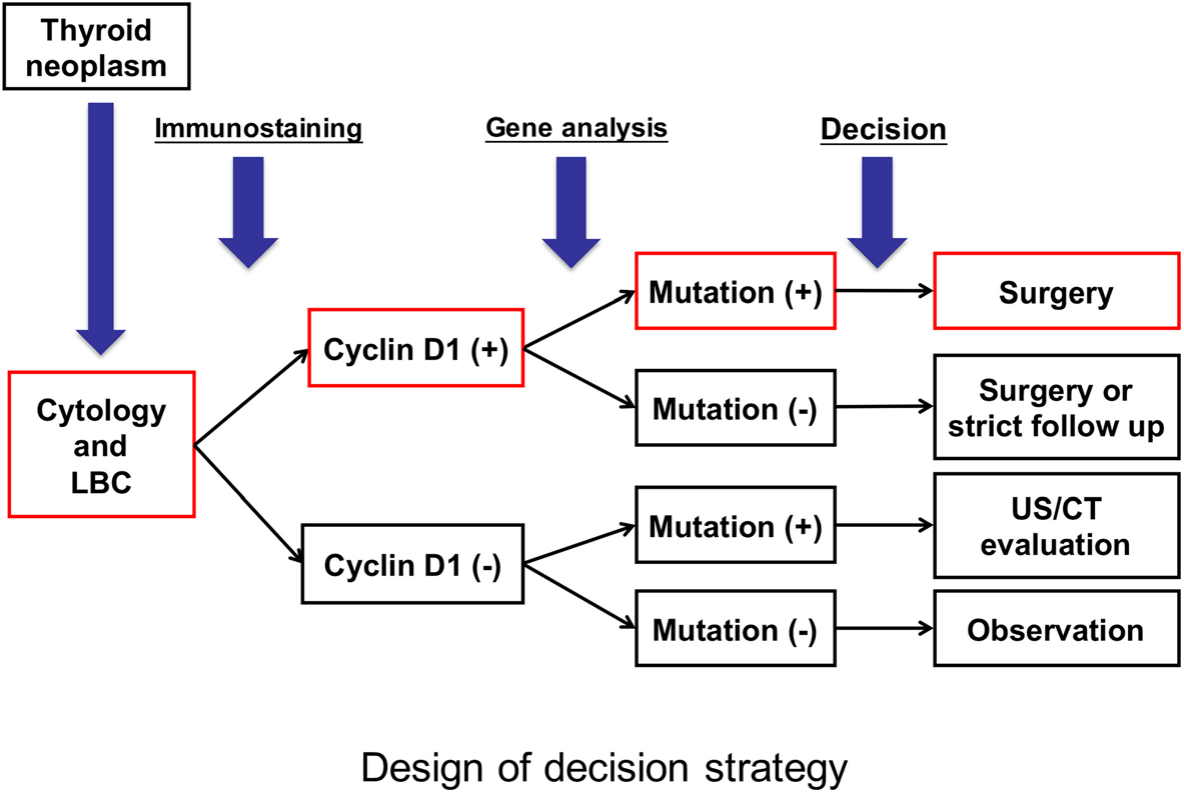
Decision strategy for thyroid neoplasm based on tumor cytology of cyclin D1 immunostaining and targeted next-generation sequencing to improve the current treatment strategy for patients. LBC, liquid based cytology

In Fig. 3, we anticipate that our proposed cytological screening system will lower medical expenses by reducing operative complications resulting in fewer unnecessary operations.

We also compared cyclin D1 immunolabeling with follicular neoplasm. A FNA biopsy in patients with a follicular adenoma or carcinoma is characterized by abundant follicular epithelial cells in sheets with crowding and overlapping of cells, microfollicle formation, and scant or no colloid [17]. Although there was no significant difference between follicular carcinoma and adenoma, the percentage of cyclin D1 immunolabeled tumor cells tended to be higher in the follicular carcinoma. Nevertheless, follicular tumors tended to have low PPV from cyclin D1. Low PPV of cyclin D1 immunostaining for follicular tumors was related to low atypia of nuclei, low cell proliferative activity, and variation in follicular neoplasms. Capsular invasion and/or vascular invasion are required for the diagnosis of encapsulated follicular carcinoma and the difference with adenoma can only be made by histological but not cytological analysis. However, cytological analysis may be improved by combining NGS of targeted genes such as TP53, PAX8-PPAR and RAS.

However, the low number of available retrospective cases limits our evaluation of the utility of this system. BG cases have higher median staining ratios of Ki67 than AG tissues, and the MSR of BG cases is similar to those of every tumour analysed. We thought this was due to an increase in inflammatory lymphocytes. Lymphocytes exhibit a well-known phenomenon that their Ki67 immunolabeling rate is high, and not that of the tumour itself [26]. We also thought this is not a problem because the thyroid follicular epithelium is recognized and evaluated for histological morphology but was limited in our evaluation. In addition, there are challenges with DNA extraction and cell stability stemming from LBC. Although further testing with a larger number of patients and sufficient samples for cytological testing and genetic analysis is warranted, we believe that our proposed system for cytological diagnosis of thyroid nodules confers the advantage of not requiring sample fixation and its potential use in clinical settings may influence policy decision-making.

## Conclusion

Fine-needle aspiration is the routine method for diagnosing thyroid nodules; however, some of undetermined significance can confound efforts to identify malignancies. Our Cytologic Cyclin D1 Screening System enables rapid determination of personalized treatment strategies for patients through next generation sequencing using cytological samples highly sensitive and specificity diagnosis method.

## Additional file

Additional file 1: **Table S1**. First round PCR. (XLSX 11 kb)

## Abbreviations

AG: adenomatous goiter
ATC: anaplastic thyroid carcinoma
AUC: area under the ROC curve
AUS/FLUS: atypia of undetermined significance/follicular lesion of undetermined significance
BG: backgrounds
FA: follicular adenoma
FC: follicular carcinoma
FFPE: Formalin-fixed paraffin-embedded specimens
FNA: fine-needle aspiration
LBC: liquid based cytology
MSR: median stain ratio
MTC: medullary thyroid carcinoma
PDC: poorly differentiated thyroid carcinoma
PPV: positive predictive value
PTC: papillary thyroid carcinoma
ROC: receiver operating characteristic
WP: well-differentiated tumor with uncertain malignant potential
